# Supplementary material for: Small soluble α-synuclein aggregates are the toxic species in Parkinson’s disease
Source: Nat Commun. 2022 Sep 20;13:5512. doi: 10.1038/s41467-022-33252-6 (PMC9489799; doi:10.1038/s41467-022-33252-6)
Supplement: Supplementary file 1 — Supplementary Information [file 41467_2022_33252_MOESM1_ESM.pdf]

Supplementary Information

Small soluble α-synuclein aggregates are the toxic species in Parkinson's disease

Derya Emin[1,2], Yu P. Zhang[1,2], Evgeniia Lobanova[1,2], Alyssa Miller[1], Xuecong Li[3,4], Zengjie Xia[1,2], Helen Dakin[1,2], Dimitrios I. Sideris[1], Jeff Y. L. Lam[1,2], Rohan T. Ranasinghe[1], Antonina Kouli[5], Yanyan Zhao[6], Suman De[1,7], Tuomas P.J. Knowles[1], Michele Vendruscolo[1], Francesco S. Ruggeri[1,3,4], Franklin I. Aigbirhio[6], Caroline H. Williams-Gray[5] and David Klenerman[1,2*]

[1] Yusuf Hamied Department of Chemistry, University of Cambridge, Lensfield Road, Cambridge, UK
[2] UK Dementia Research Institute, University of Cambridge, Cambridge, UK
[3] Laboratory of Organic Chemistry, Stippeneng 4, 6703 WE, Wageningen University & Research, Netherlands
[4] Physical Chemistry and Soft Matter, Stippeneng 4, 6703 WE, Wageningen University & Research, Netherlands
[5] John van Geest Centre for Brain Repair, Department of Clinical Neurosciences, University of Cambridge, Cambridge, UK
[6] Molecular Imaging Chemistry Laboratory, Wolfson Brain Imaging Centre, Department of Clinical Neurosciences, University of Cambridge, Cambridge, UK
[7] Sheffield Institute for Translational Neuroscience, University of Sheffield, UK

*corresponding author: Prof Sir David Klenerman, email: dk10012@cam.ac.uk.

Supplementary Figures

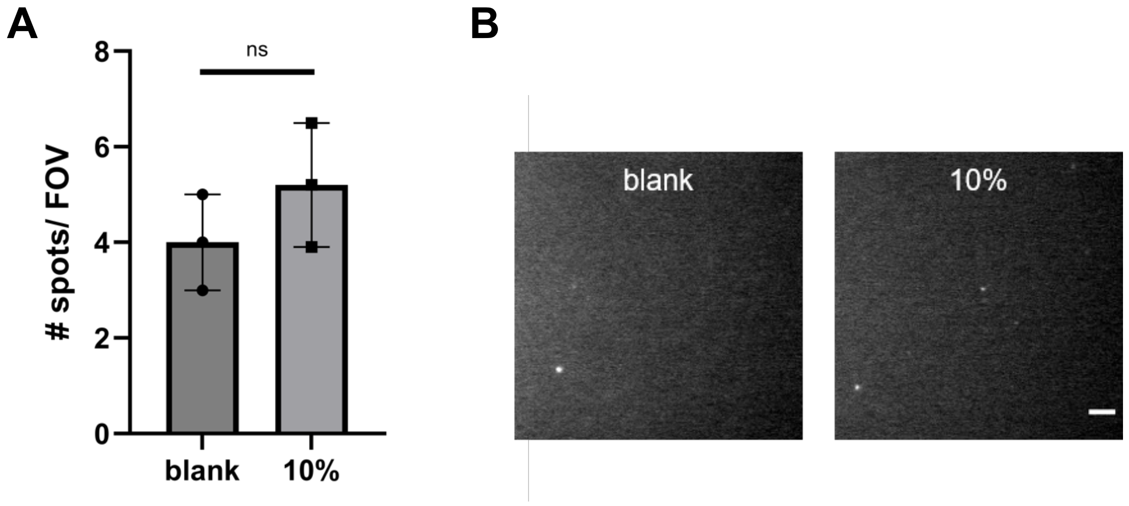

Supplementary Figure 1 Comparison of number of detected spots between negative control and 10% sucrose fraction using thioflavin T. (A) Bar graph comparing the number of detected spots per field of view between the blank (PBS) and the 10% sucrose fraction. Mean ± STD from three different replicates. Each data point is representing one replicate. Two-tailed unpaired t-test with p=0.239, N=3. **(B)** Representative TIRF images imaged with 5 μM of thioflavin T. Images are cropped and contrast adjusted. Scale bar= 5 μm. Images were representative across experiments. Source data are provided as a Source Data file. Abbreviation #= Number, FOV= Field of view.

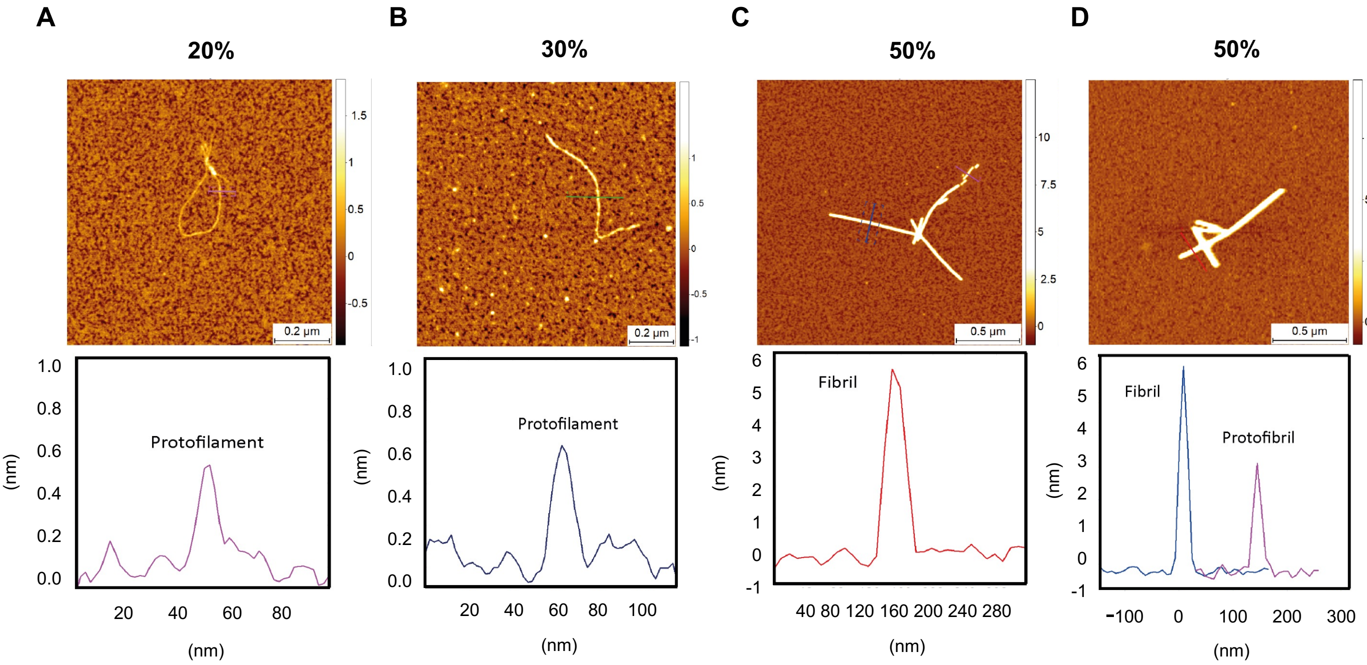

Supplementary Figure 2 Comparison between fibrils and protofilaments using AFM. Representative figures for **(A)** 20%, (B) 30% and **(C)** and **(D)** 50% sucrose fractions including the corresponding cross-sectional diameters. Images were representative across experiments. Cross-sectional diameters were generated from the provided representative figures using SPIP software and exported as a whole image.

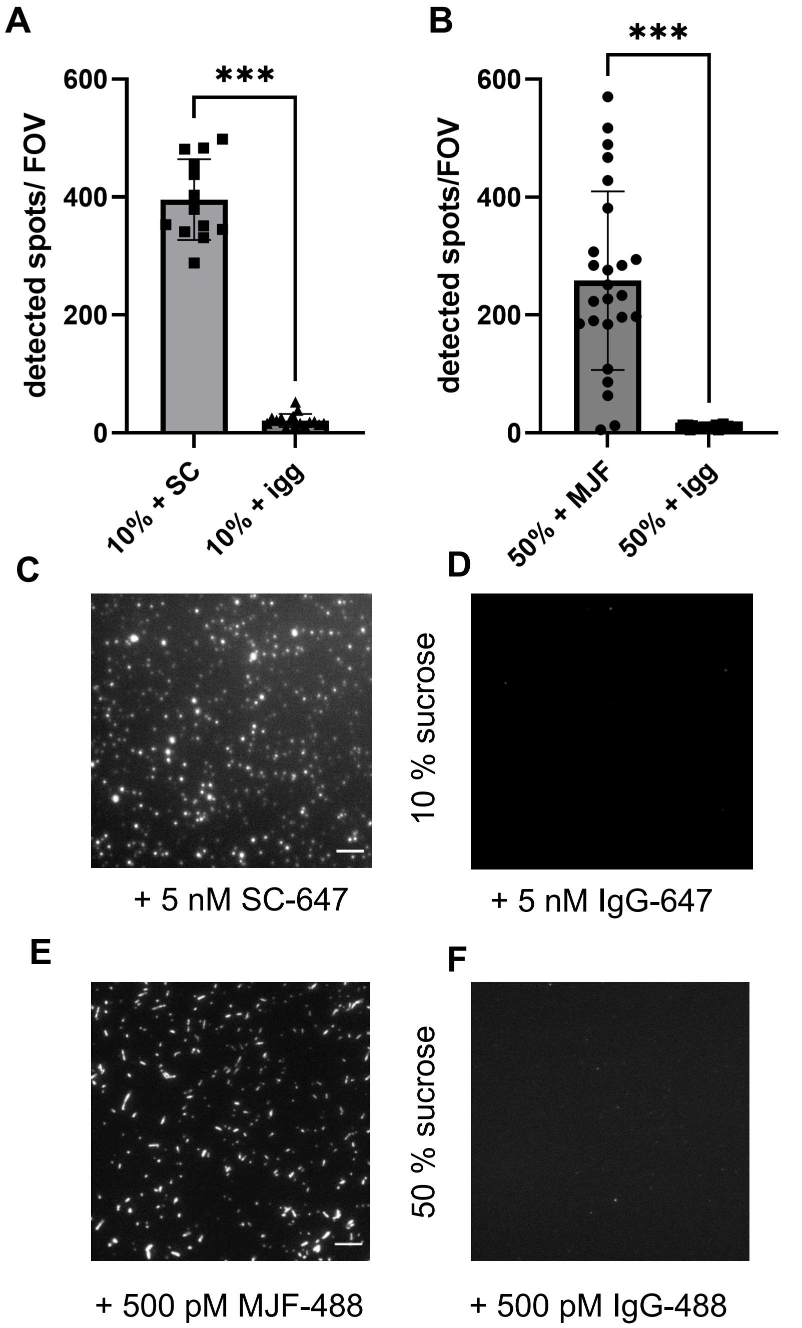

Supplementary Figure 3 Control experiments for specific binding. (A) Number of detected spots for 10% sucrose fraction imaged with SC-647 (10% + SC, N= 13 fields of view) and with isotype control IgG (10% + igg, N= 16 fields of view). Error bars are mean ± STD from different field of views. Two-tailed unpaired t-test for statistical testing with p<0.001. Each point represents one individual field of view. **(B)** Number of detected spots for 50% sucrose fraction imaged with MFJ-488 (50% + MJF, N= 25 fields of view) imaged with isotype control IgG (50% + igg, N= 25 fields of view). Error bars are mean ± SD from different field of views. Two-tailed unpaired t-test for statistical testing with p<0.001. Each point represents one individual field of view.**((C) (D)** Representative TIRF images from the fraction imaged with SC antibody and IgG control. Images are contrast adjusted. Scale bar= 5μm. **(E) (F)** Representative TIRF images from the fraction imaged with MJF antibody and IgG control. Images are contrast adjusted. Scale bar= 5μm. Images were representative across experiments. Source data are provided as a Source Data file. FOV= field of view, MJF= MJFR-14-6-4-2 antibody, SC= Santa cruz 211 antibody, IgG= Isotype control, 488= Alexa Fluor 488 labelled antibody, 647= Alexa Fluor 647 labelled antibody, nM= Nanomolar, pM= Picomolar. Details about the antibodies can be found in SI Table 3.

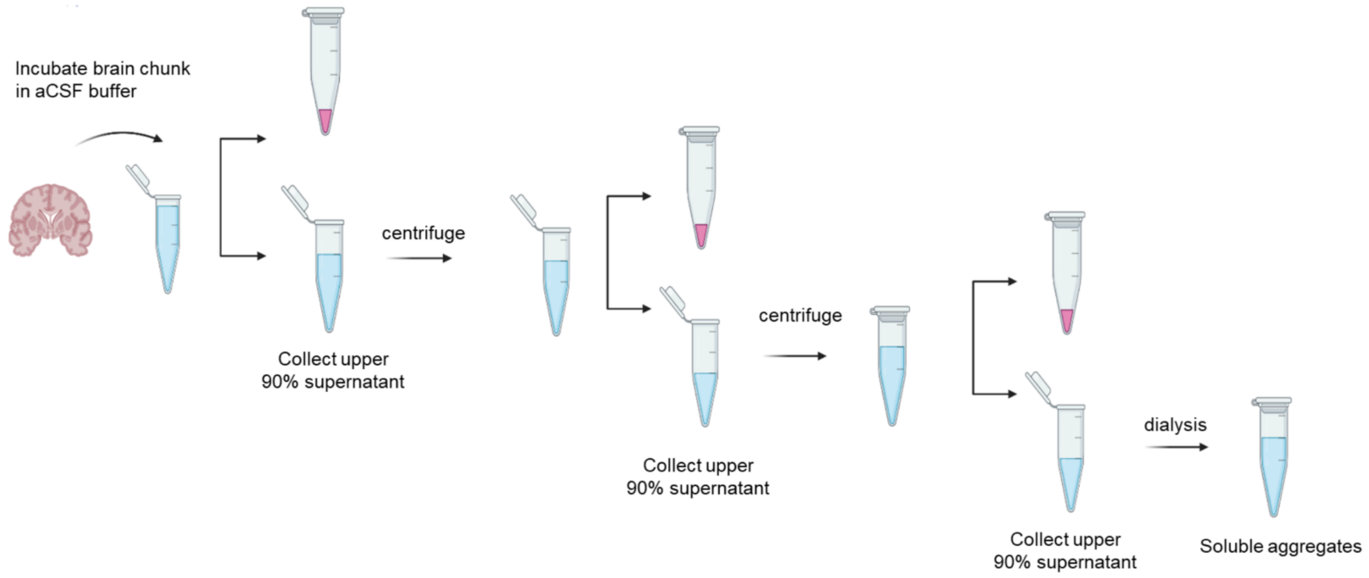

Supplementary Figure 4 Schematic showing the workflow of aggregate extraction from brain samples. The brain is cut into one big piece (300 mg), incubated in buffer for 1.5 h, afterwards only the upper 90% are centrifuged. After centrifugation, the remaining upper 90% are centrifuged again. In the last step the samples are dialysed for 72 h. The schematic has been created with BioRender.com.

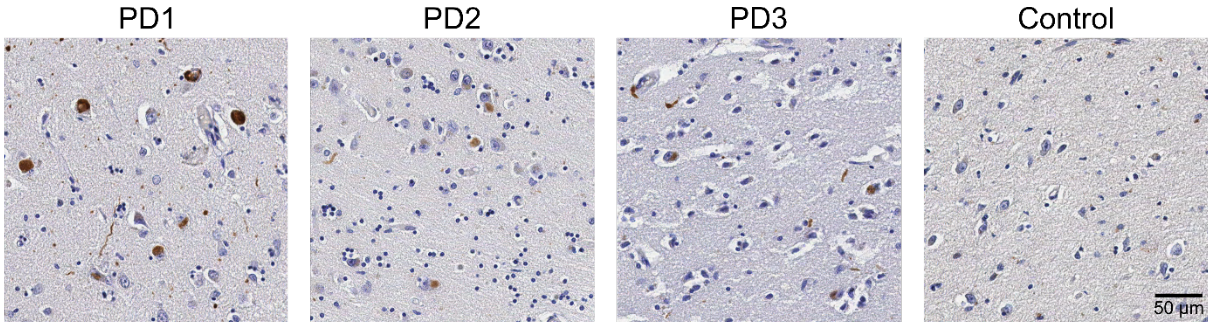

Supplementary Figure 5 Immunohistochemical staining of post-mortem tissue for α-synuclein pathology. Representative images showing α-synuclein positive Lewy bodies and Lewy neurites in the amygdala in the 3 PD cases. No alpha synuclein pathology was observed in the control brains (one representative example shown). Images were representative across experiments. PD= Parkinson's patients.

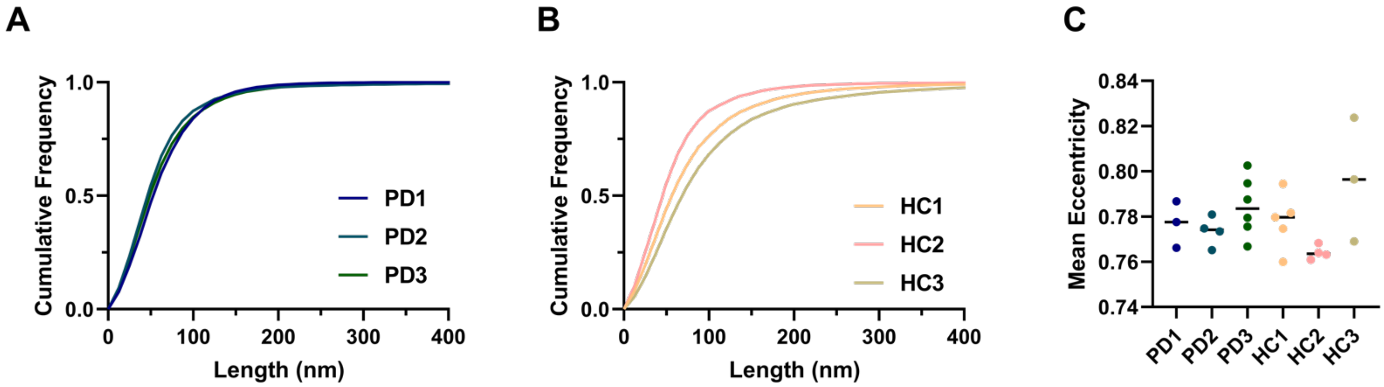

Supplementary Figure 6 Length and Eccentricity individual patient data. (A) Cumulative Frequency distribution of aggregates detected with AD-PAINT in PD brain extracts. The graph consists of the collected data from at least three replicates for each patient. **(B)** Cumulative Frequency distribution of aggregates detected with AD-PAINT for control brain extracts. The graph consists of the collected data from at least three replicates for each patient. **(C)** Scatter plot for mean eccentricity for each individual patient. Each dot represents one replicate. The horizontal line is indicating the median. Source data are provided as a Source Data file. PD= Parkinson's patients, HC= controls.

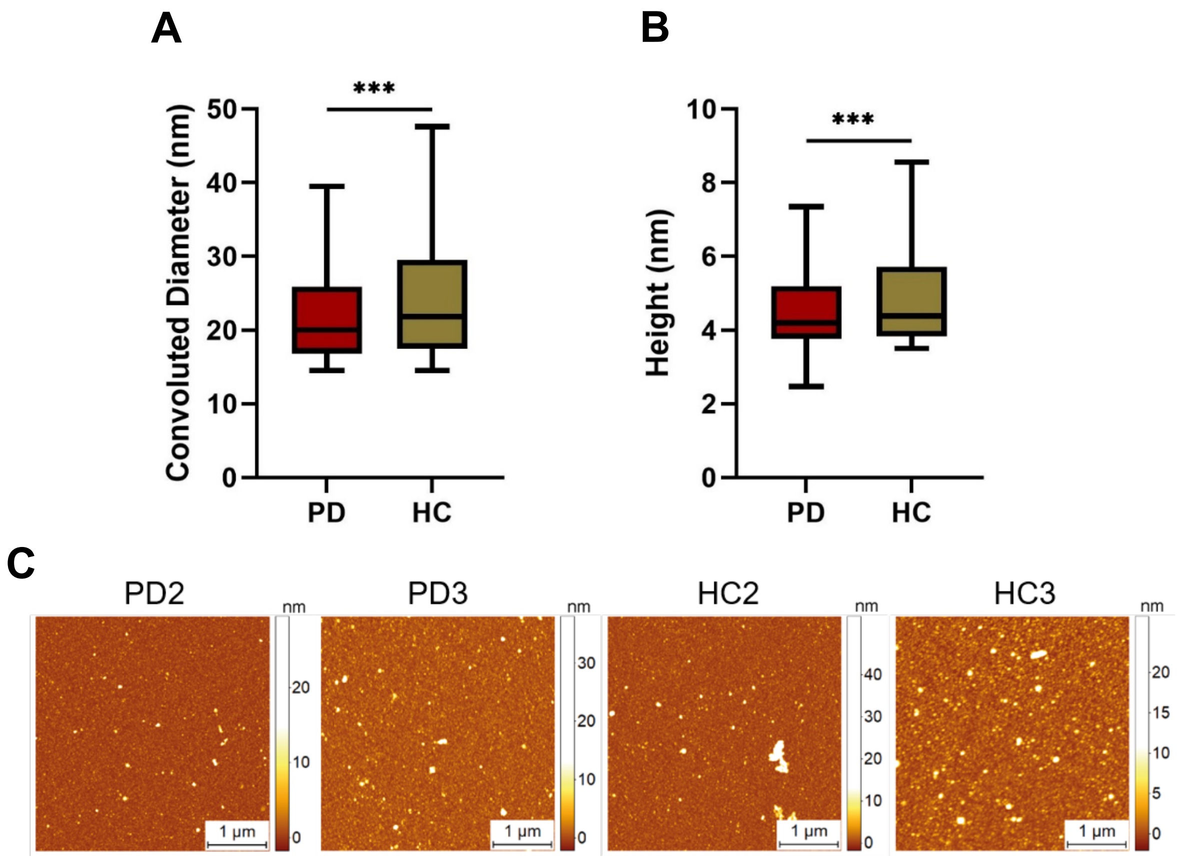

Supplementary Figure 7 AFM images of aggregates in PD and control brain. Distribution of **(A)** diameter and of cross-sectional **(B)** height of the aggregated species present in the PD (burgundy) vs control (beige) cohort. Tukey box limit extends from the 25th-75th quartile with the horizontal line in the centre indicating the median. The whiskers stretch from 25th quartile -1.5 IQR and 75th quartile + 1.5 IQR. Median diameter ± SEM for the aggregates abundant in PD patients corresponds to 20 ± 1 nm (N=48800) and average height 4.2 ± 0.05 nm (N=47974). Control samples have a median diameter of 22 ± 1 nm (N=43395) and height of 4.4 ± 0.05 nm (N=31211). An area larger than 200 μm^2 was randomly sampled for each sample. The data represents the collected distribution from three brains per group. Mann-Whitney t-test with $p<0.001$. **(C)** High-resolution 3-D map representative images of two PD patients and two controls. Source data are provided as a Source Data file. PD= Parkinson's patients, HC= controls.

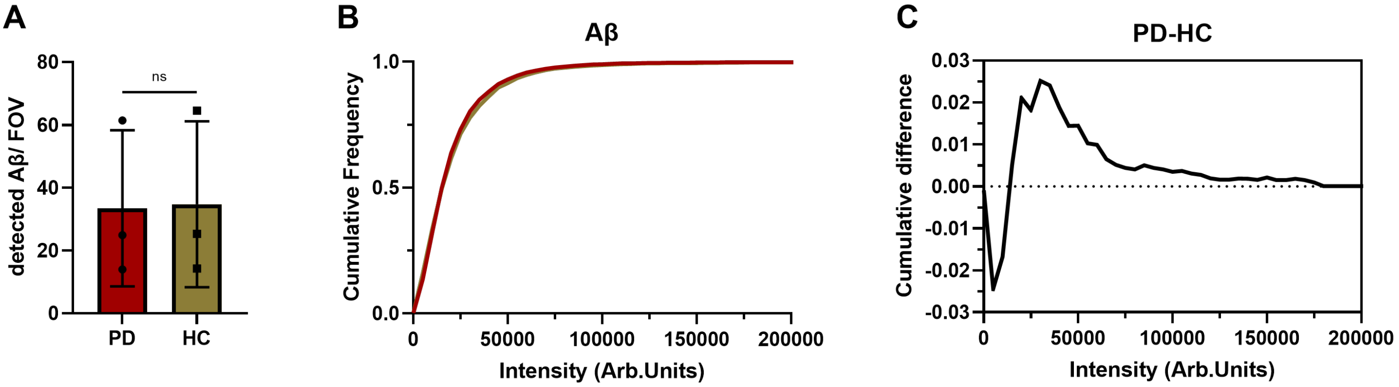

Supplementary Figure 8 SimPull analysis of extracted aggregates shows no difference in Aβ. (A) Number of detected Aβ aggregates per field of view for PD (burgundy) and controls (beige). (Two-tailed unpaired t-test, p= 0.954, N=3) Bar graphs represent mean ± STD from three cases. Each point is representing one patient**. (B)** Cumulative frequency distribution for Aβ aggregate intensity for PD (burgundy) and control (beige) measured using SimPull. Each histogram is the collected data from three cases. **(C)** Cumulative difference for Aβ aggregate intensity for PD versus controls. Source data are provided as a Source Data file. FOV= Field of view, PD= Parkinson's disease, HC= controls, Aβ= Abeta, Arb.Units= Arbitrary units.

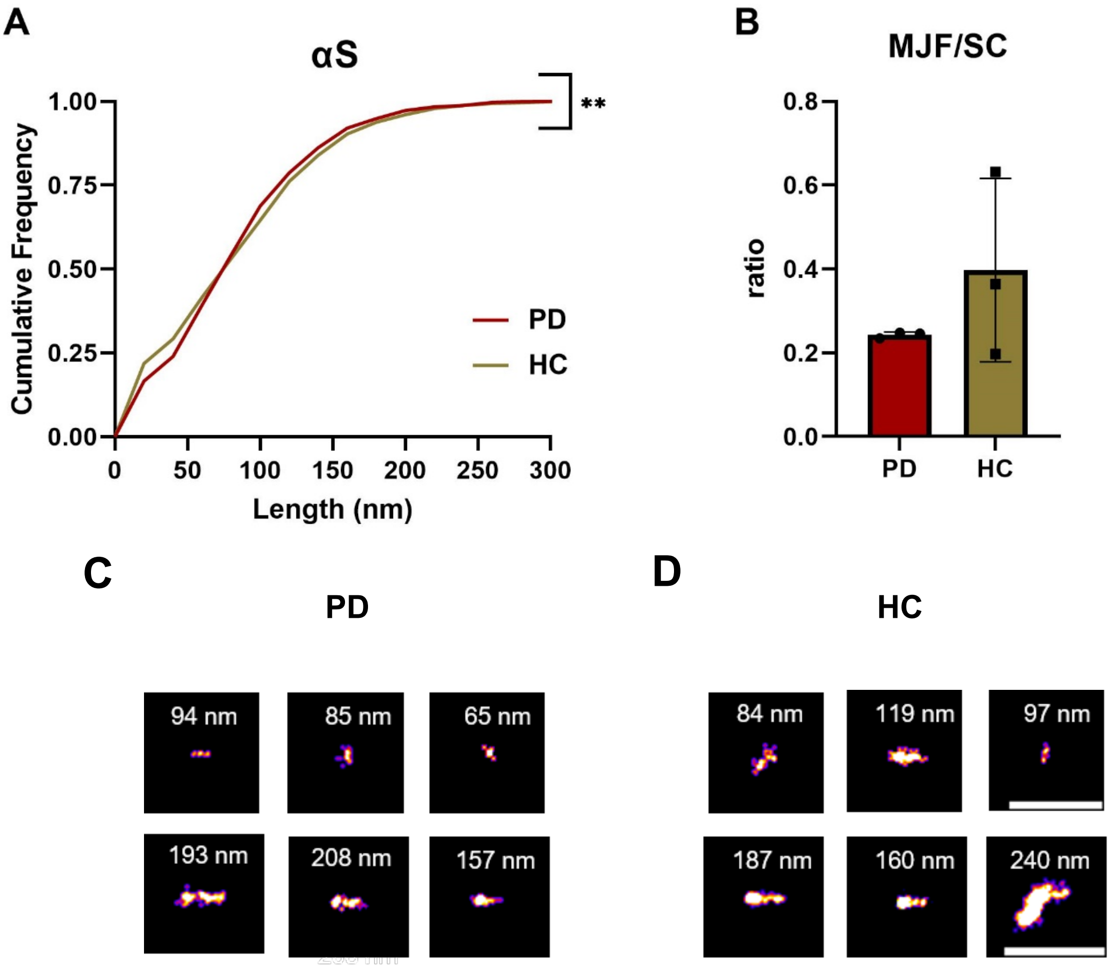

Supplementary Figure 9 Control brain samples contain more aggregated α-synuclein. (A) Length distribution from PD and controls determined by SiMPull-STORM using the MJF antibody. (Kolmogorov-Smirnov test, p=0.002, N=3) **(B)** Ratio of detected spots using the MJF and SC antibody, respectively. Bar graphs represent mean ± STD from three cases. Each individual data point represents one of the patients. (**C**) Example images of aggregates from PD patients. (**D**) Example images of aggregates from controls. Scale bar= 500nm. Source data are provided as a Source Data file. PD= Parkinson's disease, HC= controls, MJF= MJFR-14-6-4-2 antibody, SC= Santa cruz 211 antibody. Details about the antibodies can be found in SI Table 3.

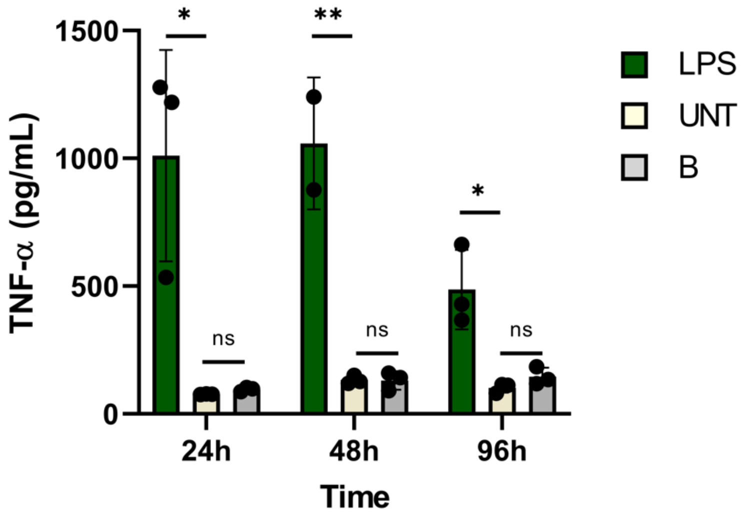

Supplementary Figure 10 Control data for the inflammatory assay. Controls for inflammatory assay in Figure 8E. BV2 cells were treated with positive control Lipopolysaccharide (LPS at 10 ng/mL, green) or negative control buffer (B, gray) or untreated (UNT, yellow) over the same time course as the experiments using brain samples. Each point represents one biological replicate. Error bars are mean ± STD. One-way ANOVA with Dunnett's multiple comparison test with a significance level of $\alpha = 0.05$. $p = 0.05$ for LPS vs UNT after 24h, $p < 0.001$ for LPS vs UNT after 48h, $p = 0.004$ for LPS vs UNT after 96h. Source data are provided as a Source Data file.

Supplementary Tables

Supplementary Table 1 Descriptive statistics for thioflavin T data in Figure 2.

FRACTIONS	20%	30%	40%	50%
**MEDIAN**	0.46	0.48	0.53	0.56
**25% PERCENTILE**	0.32	0.34	0.35	0.36
**75% PERCENTILE**	0.80	0.79	0.88	1.00
**NUMBER OF AGGREGATES**	3378	8296	11812	11816

Supplementary Table 2 Total protein concentration determined by BCA for each patient for PD and HC.

NO	PD		NO	HC	
	Mean (µg/mL)	STD		Mean (µg/mL)	STD
**1**	228.0167	4.142974	**1**	605.22	33.97795
**2**	484.27	16.16246	**2**	535.07	13.05415
**3**	522.4367	44.61474	**3**	944.41	66.87118

Supplementary Table 3 Overview about specifics of the antibodies used in this work.

ANTIBODY	HOST	AMOUNT	MODIFICATION	VALIDATION	SUPPLIER
**6E10**	Mouse	500 pM	647-labelled	[1–3]	Biolegend Cat. 803021
**6E10**	Mouse	10 nM	biotinylated	[1–3]	Biolegend Cat. 9340-02
**MJF**	Rabbit	500 pM	647-labelled	[4,5]	Abcam Cat. ab216309
**MJF**	Rabbit	10 nM	biotinylated	[4,5]	Abcam Cat. ab227047
**SC**	Mouse	5 nM	647-labelled	[6,7]	SantaCruz Cat. sc-12767 AF647
**SC**	Mouse	10 nM	biotinylated	[6,7]	SantaCruz Cat. sc-12767
**SA340**	Rabbit	1:250 dilution	n/a	[8]	Enzo Life Sciences Cat. BML-SA3400-0100
**ISOTYPE CONTROL**	Mouse	5 nM or 500 pM	647-labelled	Supplier website	ThermoFisher, Cat. MA-18168
**ISOTYPE CONTROL**	Rabbit	500 pM	647-labelled	[9,10]	Abcam, Cat. ab199093

References

1. Kumar, R., Nordberg, A. & Darreh-Shori, T. Amyloid-β peptides act as allosteric modulators of cholinergic signalling through formation of soluble BAβACs. *Brain* **139**, 174–192 (2016).
2. Abramowski, D. *et al.* Transgenic expression of intraneuronal Aβ42 but not Aβ40 leads to cellular Aβ lesions, degeneration, and functional impairment without typical Alzheimer's disease pathology. *Journal of Neuroscience* **32**, 1273–1283 (2012).
3. Herzig, M. C. *et al.* Aβ is targeted to the vasculature in a mouse model of hereditary cerebral hemorrhage with amyloidosis. *Nature Neuroscience* **7**, 954–960 (2004).
4. Pantazopoulou, M. *et al.* Distinct alpha-Synuclein species induced by seeding are selectively cleared by the Lysosome or the Proteasome in neuronally differentiated SH-SY5Y cells. *Journal of Neurochemistry* **156**, 880–896 (2021).
5. Sanyal, A., Novis, H. S., Gasser, E., Lin, S. & LaVoie, M. J. LRRK2 Kinase Inhibition Rescues Deficits in Lysosome Function Due to Heterozygous GBA1 Expression in Human iPSC-Derived Neurons. *Frontiers in Neuroscience* **14**, (2020).
6. Choi, I. *et al.* Microglia clear neuron-released α-synuclein via selective autophagy and prevent neurodegeneration. *Nature Communications* **11**, (2020).
7. Dansithong, W. *et al.* Generation of SNCA cell models using zinc finger nuclease (ZFN) technology for efficient high-throughput drug screening. *PLoS ONE* **10**, (2015).
8. Chen, L., Xie, Z., Turkson, S. & Zhuang, X. A53T human α-synuclein overexpression in transgenic mice induces pervasive mitochondria macroautophagy defects preceding dopamine neuron degeneration. *Journal of Neuroscience* **35**, 890–905 (2015).
9. Honjoh, K., Nakajima, H., Hirai, T., Watanabe, S. & Matsumine, A. Relationship of Inflammatory Cytokines From M1-Type Microglia/Macrophages at the Injured Site and Lumbar Enlargement With Neuropathic Pain After Spinal Cord Injury in the CCL21 Knockout (plt) Mouse. *Frontiers in Cellular Neuroscience* **13**, (2019).
10. Ligorio, M. *et al.* Stromal Microenvironment Shapes the Intratumoral Architecture of Pancreatic Cancer. *Cell* **178**, 160-175.e27 (2019).
